# Supplementary material for: Phenotypic and Genotypic Characterization of Macrolide, Lincosamide and Streptogramin B Resistance among Clinical Methicillin-Resistant Staphylococcus aureus Isolates in Chile
Source: Antibiotics (Basel). 2022 Jul 25;11(8):1000. doi: 10.3390/antibiotics11081000 (PMC9332560; doi:10.3390/antibiotics11081000)
Supplement: Supplementary file 1 [file antibiotics-11-01000-s001.zip › antibiotics-1803608-supplementary.pdf]

antibiotics

MDPI

Supplementary File

Table S1. Primers used in this study.

Gene target	Primer sequence (5′-3′)	Product size (bp)
ermA	F-TATCTTATCGTTGAGAAGGGAT<br>R-CTACACTTGGCTTAGGATGAAA	139
ermB	F- GAAAAGGTACTCAACCAAATA<br>R- AGTAACGGTACTTAAATTGTTTAC	163
ermC	F-CTTGTTGATCACGATAATTTCC<br>R-ATCTTTTAGCAAACCCGTATTC	190
msrA	F-TCCAATCATAGCACAAAATC<br>R-AATTCCCTCTATTTGGTGGT	163
pvl	F-ATCATAGGTAAAATGTCTGACATGATCCA<br>R-GCATCAASTGTATTGGATAGCAAAAGC	433
mecA	F-GTGAAGATATACCAAGTGATT<br>R-ATGCGCTATAGATTGAAAGGAT	147
SCC*mecI*	F-GCTTTAAAGAGTGTCGTTACAGG<br>R-GTTCTCTCATAGTATGACGTCC	613
SCC*mecII*	F-CGTTGAAGATGATGAAGCG<br>R-CGAAATCAATGGTTAATGGACC	398
SCC*mecII, VIII*	F-TAGCTTATGGTGCTTATGCG<br>R-GTGCATGATTTCATTTGTGGC	128
Mercury elemnt of SCC-*mecIII*	F-CCATATTGTGTACGATGCG<br>R-CCTTAGTTGTCGTAACAGATCG	280
SCCmec III and IIIA	F-TTCTCATTGATGCTGAAGCC<br>R-GTGTAATTTCTTTTGAAAGATATGG	257
SCCmec IVa	F-GCCTTATTCGAAGAAACCG<br>R-CTACTCTTCTGAAAAGCGTCG	776
SCCmec IVb, IVF	F-TCTGGAATTACTTCAGCTGC<br>R-AAACAATATTGCTCTCCCTC	493
SCCmec IVc, IVE	F-CCTGAATCTAAAGAGATACACCG<br>R-GGTTATTTTCATAGTGAATCGC	200
SCCmec IVd	F-CTCAAAATACGGACCCCAATACA<br>R-TGCTCCAGTAATTGCTAAAG	881
SCCmec IVE, IVF	F-CAGATTCATCATTTCAAAGGC<br>R-AACAACTATTAGATAATTTCCG	175
SCCmec V	F-GAACATTGTTACTTAAATGAGCG<br>R-TGAAAGTTGTACCCTTGACACC	325
SCCmec VI, VIII	F-ATCGCTCATTATGGATACYGC<br>R5-CCATTTTTTGATAACCTGAACG<br>R6- CTATTTTTTTATAGCCTGAACG	106
